# P-1737. A pilot intervention trial to reduce the use of post-procedural antimicrobials after common endoscopic urologic surgeries

**DOI:** 10.1093/ofid/ofae631.1900

**Published:** 2025-01-29

**Authors:** Daniel J Livorsi, Qianyi Shi, Steven Alberding, Knute Carter, Ryan Steinberg

**Affiliations:** University of Iowa Carver College of Medicine, Iowa City, Iowa; Iowa City VA Health System, Iowa City, Iowa; University of Iowa Department of Biostatistics, Iowa City, Iowa; University of Iowa Carver College of Medicine, Iowa City, Iowa; University of Iowa Carver College of Medicine, Iowa City, Iowa

## Abstract

**Background:**

Post-procedural antimicrobial prophylaxis is not recommended by professional guidelines but is still commonly prescribed and can lead to patient harm. The goal of this multicenter pilot intervention trial was to encourage less frequent use of post-procedural antimicrobials after common endoscopic urologic procedures.

**Table 1.**
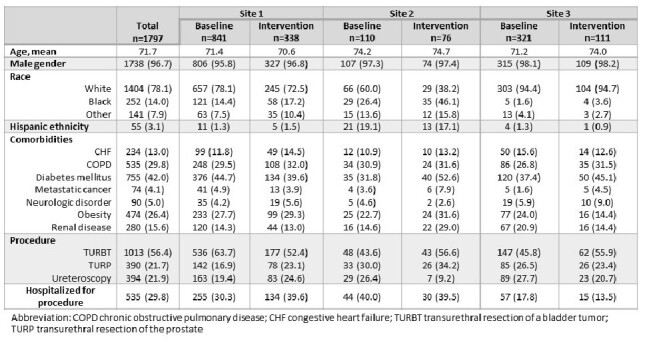
Characteristics of patients across the 3 participating hospitals during both the baseline and intervention periods

**Methods:**

We evaluated a bundled intervention using a pre-post, quasi-experimental design at three Veterans Affairs medical centers that performed common endoscopic urologic procedures: ureteroscopy and transurethral resection of a bladder tumor or prostate. There was a 2-year baseline, 1-month implementation, and 12-month intervention period. The intervention consisted of education, local champion(s), and audit-and-feedback of data, aggregated to the hospital-level, on the frequency of post-procedural antimicrobial use with comparisons to other facilities. The primary outcome was antimicrobial use on post-procedural day 1 and was evaluated using a logistic regression model for each site. Secondary outcomes were a) unplanned visits (*i.e.*, Emergency Department visits and/or hospital readmissions within 30 days of the procedure) as well as b) late antimicrobial prescriptions (*i.e.*, antimicrobials prescribed within 7-30 days after the procedure).

**Figure 1.**
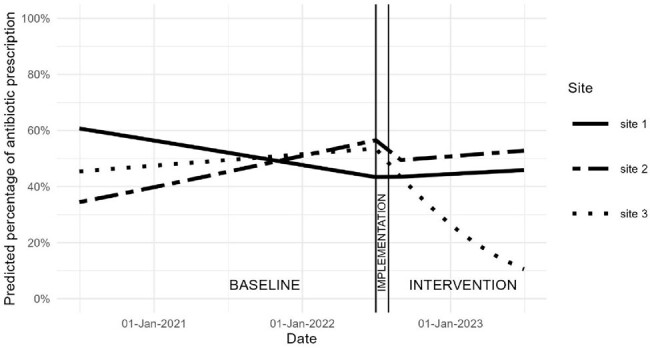
Probability of post-procedural antimicrobial use after common urologic procedures across the 3 participating hospitals during the baseline and intervention periods

**Results:**

There were 1,272 procedures performed across all sites at baseline compared to 525 during the intervention period (Table 1). During the baseline period, 644 (50.6%) patients received post-procedural antimicrobials compared to 216 (41.1%) during the intervention period. There was no change in the use of post-procedural antimicrobials at sites 1 and 2 between the two periods (Figure 1). At site 3, the odds of prescribing a post-procedural antimicrobial significantly decreased during the intervention period relative to the baseline time trend (0.089; 95% CI 0.016-0.445). There was no significant increase in unplanned visits or late antimicrobial prescriptions at any of the sites.

**Conclusion:**

Implementation of a bundled intervention was associated with reduced post-procedural antimicrobial use after urologic procedures at 1 of the 3 participating sites. These mixed findings support the safety and also the difficulty of implementing guidelines on surgical antimicrobial prophylaxis.

**Disclosures:**

**Daniel J. Livorsi, MD**, Merck: Grant/Research Support

